# A novel adomavirus from proliferative skin lesions of a broadnose sevengill shark (*Notorynchus cepedianus*)

**DOI:** 10.1038/s44298-026-00210-8

**Published:** 2026-07-02

**Authors:** Lawrence M. Gordon, Joseph L. Sevigny, Christopher B. Buck, Michael J. Murray, Inga F. Sidor, Alisa L. Newton, Scott M. Palisoul, Muriel Kelly, Adane S. Nigatu, Stephen D. Simpson, Vsevolod L. Popov, Thomas B. Waltzek, Gregory J. Tsongalis, Salvatore Frasca, W. Kelley Thomas

**Affiliations:** 1 Hubbard Center for Genome Studies, Department of Molecular, Cellular, and Biomedical Sciences, University of New Hampshire, Durham, NH USA; 2 Laboratory of Cellular Oncology, National Cancer Institute, National Institutes of Health, Bethesda, MD USA; 3 Monterey Bay Aquarium, Monterey, CA USA; 4 New Hampshire Veterinary Diagnostic Laboratory, University of New Hampshire, Durham, NH USA; 5 ZooQuatic Laboratory, LLC, Baltimore, MD USA; 6 Department of Pathology and Laboratory Medicine, Dartmouth Hitchcock Medical Center, Lebanon, NH USA; 7 Geisel School of Medicine at Dartmouth, Hanover, NH USA; 8 Department of Pathology, University of Texas Medical Branch, Galveston, TX USA; 9 Washington Animal Disease Diagnostic Laboratory, Department of Veterinary Microbiology and Pathology, College of Veterinary Medicine, Washington State University, Pullman, WA USA; 10 Department of Pathobiology and Veterinary Science, University of Connecticut, Storrs, CT USA

**Keywords:** Computational biology and bioinformatics, Diseases, Genetics, Microbiology, Molecular biology

## Abstract

In May of 2022, an aquarium-maintained broadnose sevengill shark (*Notorynchus cepedianus*) developed proliferative skin lesions that prompted pathologic and molecular investigation. Histopathologic examination revealed epidermal hyperplasia consisting of proliferation of spinous epithelial cells with mild dysplasia. Metagenomic sequencing identified a novel adomavirus with an 18,834 base pair circular double-stranded DNA genome. The virus, provisionally named broadnose sevengill shark adomavirus (7AdoV), contains two bidirectionally expressed protein-coding gene sets. Genomic annotation and structural predictions of proteins were used to contextualize 7AdoV phylogenetically and functionally. Transcriptomic analysis showed that expression of the structural late gene set was higher than the replicative early gene set at the time of diagnostic sampling. In situ hybridization using RNAscope technology localized transcripts of the adomavirus Wasp gene to epithelial cells of the hyperplastic epidermis. Infection by this novel adomavirus was associated with superficial and proliferative lesions that were self-limiting and resolved in this shark.

## Introduction

The broadnose sevengill shark (*Notorynchus cepedianus*) is a large (up to three meters total length) demersal shark with a cosmopolitan diet that includes marine mammals, other elasmobranchs, teleosts, and invertebrates (cephalopod and gastropod mollusks and crustaceans)^1^. The species has a patchy but global distribution in the temperate, nearshore waters of the Atlantic and Indo-Pacific oceans^2^ and in coastal estuaries of the Pacific Northwest^3^. Over the last 65 years, the global population is suspected to have undergone a 30–49% reduction, gaining the shark vulnerable status by the International Union for the Conservation of Nature^4^. Like other shark species, targeted and incidental capture in commercial and recreational fisheries are their primary conservation threat; however, the species’ preference for nearshore habitats and depths of less than two meters puts them at particular risk^4^. Currently, populations of this species are not managed throughout their extant range.

Elasmobranchii, the taxonomic group of cartilaginous fish that includes sharks, rays, skates, and sawfish^5^, represents the oldest phylogenetic group of vertebrates with all components necessary for an adaptive immune response^6^. Although viral diseases affecting elasmobranchs have been previously reported, the host range and symptomatic extent of these are not yet well-defined^7^. Our understanding of broadnose sevengill sharks is limited^8,9^. Among DNA viruses in elasmobranchs, one herpesvirus^7^, one papillomavirus^10^, two polyomaviruses^11,12^, and two adomaviruses^13,14^ have been molecularly confirmed. Suspected cases of adenovirus^15^ and iridovirus^15,16^ have been reported based on virion morphology. Recent surveys of elasmobranch tissue archives have also revealed diverse RNA viruses infecting a range of elasmobranch hosts^17^.

The *Adomaviridae* is an emerging family of circular double-stranded DNA viruses named for their similarities to adenoviruses, polyomaviruses, and papillomaviruses^18^. Initial discoveries date back to 2011, when the viral agent associated with endothelial cell necrosis (VECNE) in endangered Japanese eels (*Anguilla japonica*) was isolated and sequenced. VECNE is thought to have affected farmed Japanese eel populations since at least the 1980s^19^. Electron microscopy showed the virus to have adenovirus-like virions, while sequencing revealed a large open reading frame with similarity to polyomavirus large T-antigens (LT). This Japanese eel endothelial cell-infecting virus (JEECV) was hypothesized to have arisen through a chimeric recombination between an ancestral adenovirus and an ancestral polyomavirus^18,19^. In 2015, a similar virus was reported from marbled eels (*Anguilla marmorata*) collected in Taiwan. Amplification and phylogenetic analysis of the LT domain of the marbled eel adomavirus (MEAdoV) showed that it was most closely related to JEECV^20^. Full sequencing of MEAdoV revealed nine additional proteins—including a homolog of archaeal-eukaryotic primases, an adenain-like protein, five capsid proteins, and two unknown proteins—each displaying highest sequence similarity to its JEECV counterpart^21^. Sequence identity among syntenic genes, as well as remote similarity to adenovirus-like gene clusters and capsid proteins supported consideration of these eel viruses as members of a unique virus family^18^.

Further adomavirus discoveries in 2018 in the giant guitarfish (*Rhynchobatus djiddensis*), Amazon red discus (*Symphysodon discus*), and green arowana (S*cleropages formosus*) suggested subdivisions within the adomavirus family^13,18^. Replicase homologs in the guitarfish adomavirus (GAdoV) and discus adomavirus showed lower sequence similarity to polyomavirus LT proteins than those of JEECV, MEAdoV, and arowana adomavirus. These divergent replicases instead showed structural similarity to the papillomavirus E1 replicase protein while phylogenetically forming a distinct clade between LT, E1, and parvovirus NS1 proteins^18^. Based on these differences, adomaviruses were preliminarily divided into alpha (E1-replicase-like-encoding) and beta (LT-replicase-like-encoding) groups^18^. Concurrent database mining efforts expanded the known host range of adomaviruses beyond fish to amphibians and reptiles^18^.

In 2024, Buck et al. undertook a comprehensive database mining effort to identify eukaryotic DNA viruses in publicly available sequencing datasets^22^, identifying new adomaviruses in previously recognized host groups and expanding host range to include mollusks and ribbon worms^22^. Their findings unveiled evidence of widespread horizontal gene transfer between eukaryotic viruses, and broadly defined the adomaviruses as circular genomes between 9000 and 20,000 base pairs (bp) in length encoding a replicase, a Penton, and a Cah gene^22^. This study also revised the naming of adomavirus subfamilies, moving to a more descriptive convention: RepE1 for the previously known alpha adomaviruses and RepLT for the previously known beta adomaviruses. In addition, a third type of adomavirus was identified with replicases resembling those of circular, Rep-encoding ssDNA viruses (cressviruses), and termed CressRep^22^. Cressviruses are proposed to have been the evolutionary ancestor to papillomaviruses and polyomaviruses through parvovirus intermediates^23^. Together, these efforts provide a convention for identifying and classifying adomaviruses based on their gene content, genome structure, and replicase phylogeny.

In May of 2022, a juvenile male broadnose sevengill shark maintained in a commercial aquarium developed a series of proliferative skin lesions of unknown etiology. Noting the potentially infectious nature of the lesions and recalling similar infections in past sevengill sharks, the veterinary team at the aquarium began a diagnostic investigation. This report describes the use of genomics and transcriptomics to characterize and contextualize a novel RepE1 adomavirus identified from the proliferative skin lesions of that broadnose sevengill shark. Genomic data was used to predict and characterize viral proteins, while transcriptomic sequencing of ribosome-depleted RNA was used to non-selectively capture functionally relevant RNA^24^ and provide a snapshot of a RepE1 adomavirus infection trending towards resolution. This resulted in the identification and characterization of a novel adomavirus localized by in situ hybridization using RNAscope technology to the lesioned skin of the sevengill shark. This finding contributes to the range of elasmobranchs with demonstrable susceptibility to the *Adomaviridae*, as well as the trend of RepE1 adomaviruses resulting in superficial, proliferative, and self-limiting lesions in elasmobranch hosts.

## Results

### Disease presentation and histological findings

**Fig. 1:**
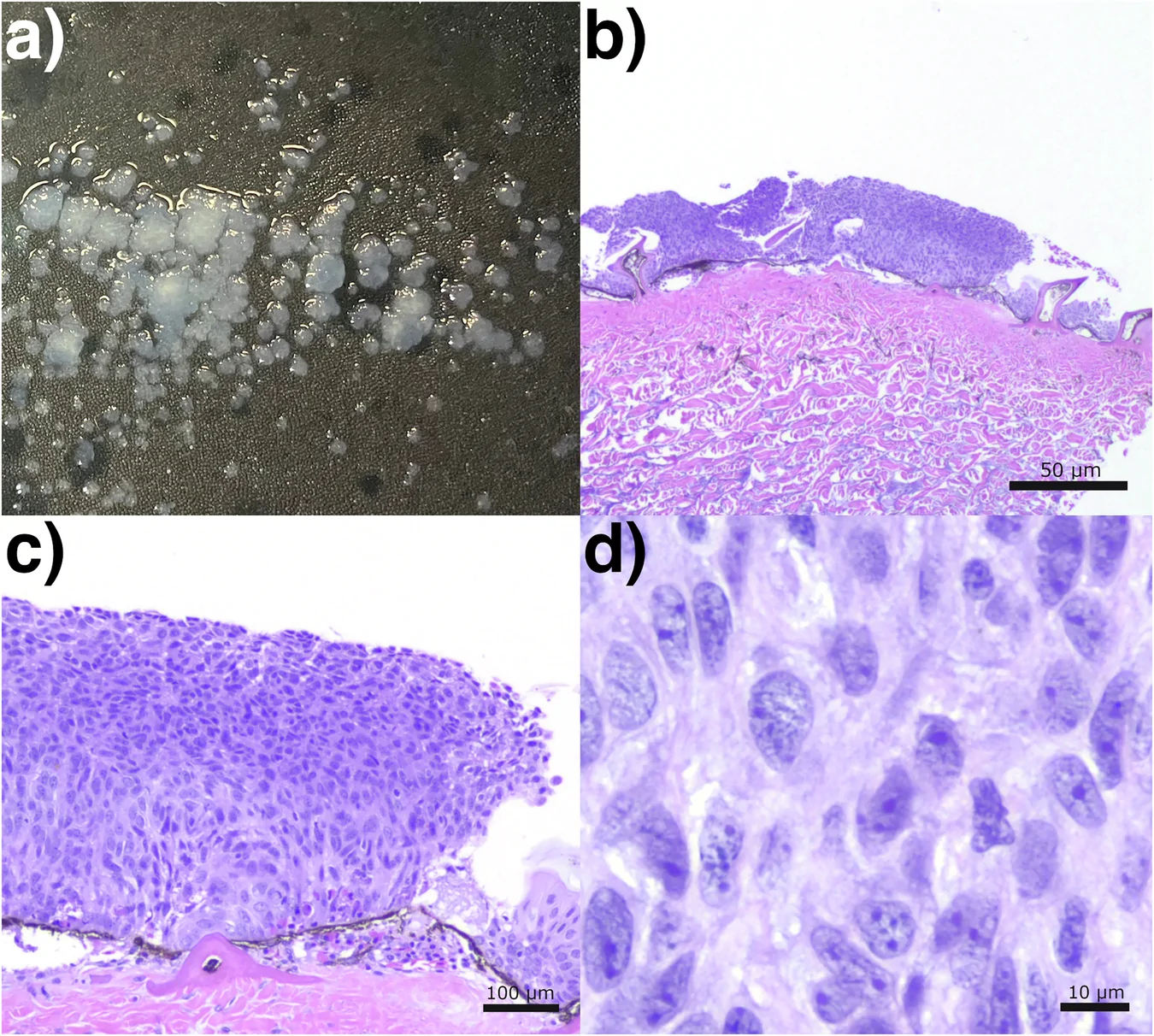
Gross and histological presentation of lesioned skin in a broadnose sevengill shark with molecular detection of 7AdoV. **a** Gross appearance of the skin of the trunk with multifocal and coalescent, raised, white to gray plaques and papules. **b** Histologic section of a plaque, which consists of a focus of marked epidermal hyperplasia that extends over the surface of placoid scales, with a flat surface and an abrupt transition to the non-lesioned epidermis (right edge of section). **c** The hyperplastic epidermis is markedly widened and mildly dysplastic, consisting of many, poorly delineated, and closely packed layers of squamous epithelial cells. **d** High magnification view of squamous epithelial cells within a focus of epidermal hyperplasia with dysplasia; cells have indistinct cytoplasmic margins, lightly eosinophilic to amphophilic cytoplasm, and a large, oval to elongate nucleus with dispersed chromatin and one to several prominent nucleoli (**b**–**d**). Hematoxylin and eosin stain, scale bars indicate size.

After ~8 months of aquarium care, lesions were noted on the skin dorsal and lateral to the left pectoral fin as a cluster of multiple, raised, white to gray, discrete and coalescing plaques and papules varying from 2 mm to several centimeters (Fig. 1a). Skin scrapings did not reveal evidence of ectoparasitism. Histological examination of grossly lesioned skin revealed focally extensive epidermal hyperplasia consisting of proliferation of spinous epithelial cells with mild dysplasia (Fig. 1b–d). Epidermal hyperplasia was accompanied by mild granulocytic infiltrates limited to the epidermis and necrosis of scattered individual spinous epithelial cells. Approximately two months after the initial emergence of lesions, biopsies of lesioned skin were repeated alongside biopsies of non-lesioned skin and blood collection. Histopathological findings in lesioned skin from the second biopsy event had the same pathological features as the original biopsy which prompted metagenomic sequence-based investigation. Lesions resolved five months after initial presentation without reappearance.

### Metagenomic sequencing identifies a novel adomavirus in lesioned skin

Whole-genome shotgun metagenomic sequencing was performed to identify non-host sequences that might represent etiological agents of epidermal hyperplasia in lesioned skin. Sequencing of four tissue samples and two blood samples generated 149,584,354 paired-end reads. Reads were mapped to the small-spotted catshark genome for host-depletion, removing 62% of the trimmed read pairs. The combined host-depleted reads were assembled and resulting contigs were compared to the nucleotide database. Most contigs were taxonomically assigned to various shark orders including Carcharhiniformes, Squaliformes, and Lamniformes indicating large amounts of host DNA remaining in the assembly.

This search also revealed two distinct alignments between an 18,859 bp de novo contig and *Rhynchobatus djiddensis* adomavirus 1 isolate UGA1 (accession NC_040625), the previously described GAdoV, a 21,537 bp circular double-stranded DNA RepE1 adomavirus affecting the giant guitarfish^13^. The first alignment shared 67.8% nucleotide identity across a 298 bp region, while the second shared 65.6% identity across a separate 320 bp region. Both alignments corresponded to regions within the EO1 locus of the guitarfish adomavirus genome, which codes for its papillomavirus-like superfamily 3 helicase (RepE1, accession YP_009552707). Further translated nucleotide-protein similarity searches with BLASTx revealed multiple alignments to other adomavirus genes and genomes across the de novo contig. Screening with CenoteTaker3^25^ and CheckV^26^ also identified this same contig as the only high-quality and only complete viral sequence in the assembly.

**Fig. 2:**
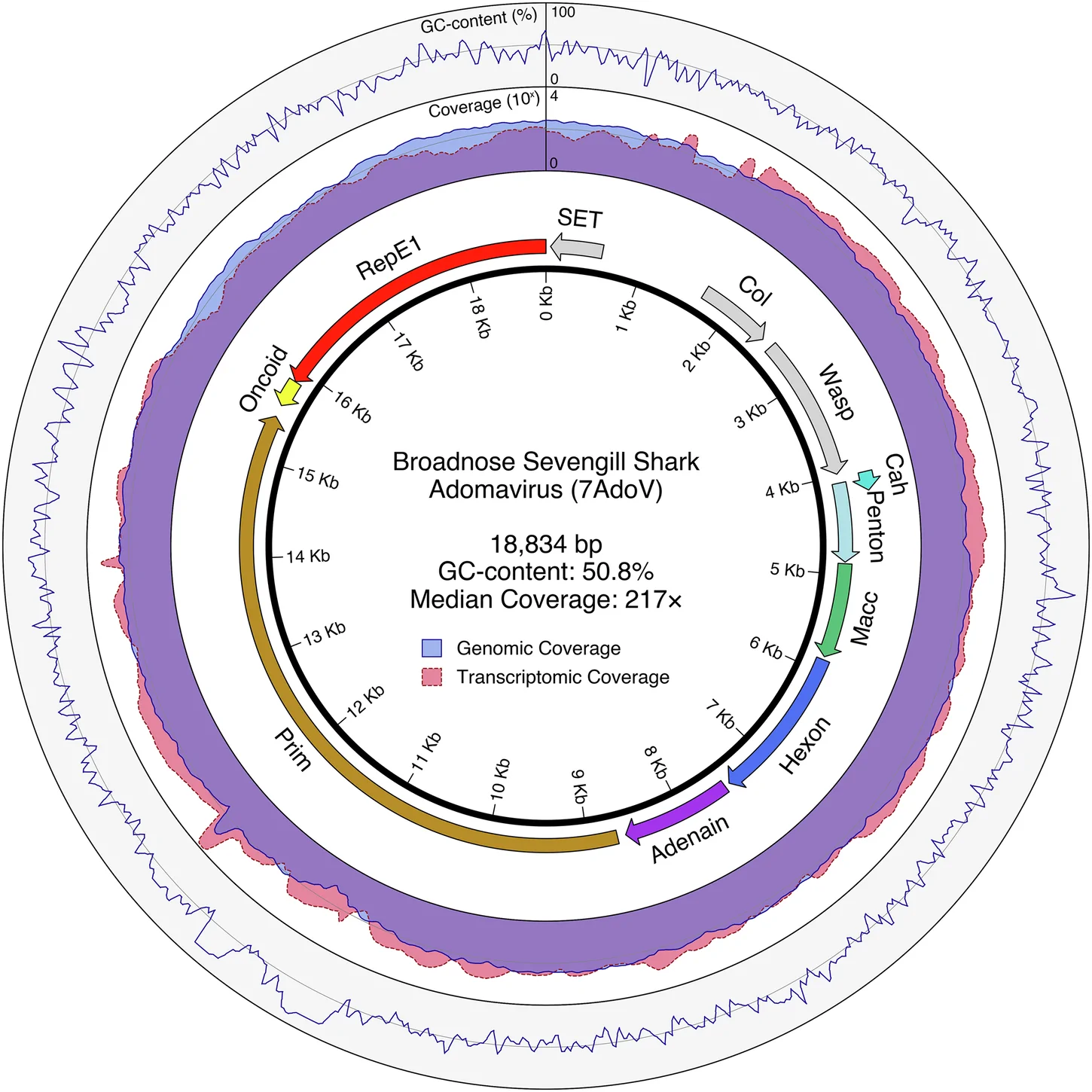
The broadnose sevengill shark adomavirus genome. 7AdoV is 18,834 bp in length with a GC-content of 50.8% and an average coverage of 217×. The eleven detected ORFs are annotated on the innermost ring using structural and sequence homology searches or synteny to prior adomaviruses (Table 2). The middle ring shows genomic and transcriptomic coverage across the genome on a log_10_ scale, and the outer ring indicates GC content. Both coverage and GC content were calculated using a 37 bp sliding window with an 18 bp step size.

The de novo contig, now assumed to be a putative adomavirus genome, was polished and circularized with additional long-read sequencing, resulting in a complete genome 18,834 bp in length with a GC-content of 50.8% and an average coverage of 217× with short-read coverage ranging from 12 to 1003×. (Fig. 2). Annotation of the broadnose sevengill shark adomavirus (7AdoV) genome revealed eleven hypothetical open reading frames (ORFs), and protein similarity searches of these ORFs using BLASTp showed similarity to GAdoV LO4 through LO8, EO2-4, EO1, and SET, leaving three unannotated ORFs. DNA and RNA reads from each sample were separately mapped to the 7AdoV genome and were only present in lesioned dorsal samples (Table 1). Searches with Pebblescout^27^ did not identify 7AdoV in public nucleotide databases, although tblastn of 7AdoV proteins against elasmobranchs did identify a candidate adomavirus from an ocellate spot skate assembly^28^ (*Okamejei kenojei*, accession JBFCOM010000348).

**Table 1:** Presence of 7AdoV by sample

Sample	DNA	Ribo-depleted RNA
Right Ventral (6774-1)	0.00	0.00
Left Ventral (6774-2)	0.00	0.00
Left Dorsal (6774-3)	54.38	6.18
Left Dorsal (6774-4)	497.74	529.19
Buffy Coat	0.00	0.00
Whole Blood	0.00	0.00

Trimmed reads per million mapping to the repeat-masked 7AdoV genome. Lesioned dorsal tissues are the only samples with adomavirus reads present.

### Folding prediction and structural searches illuminate possible functions of 7AdoV proteins

**Fig. 3:**
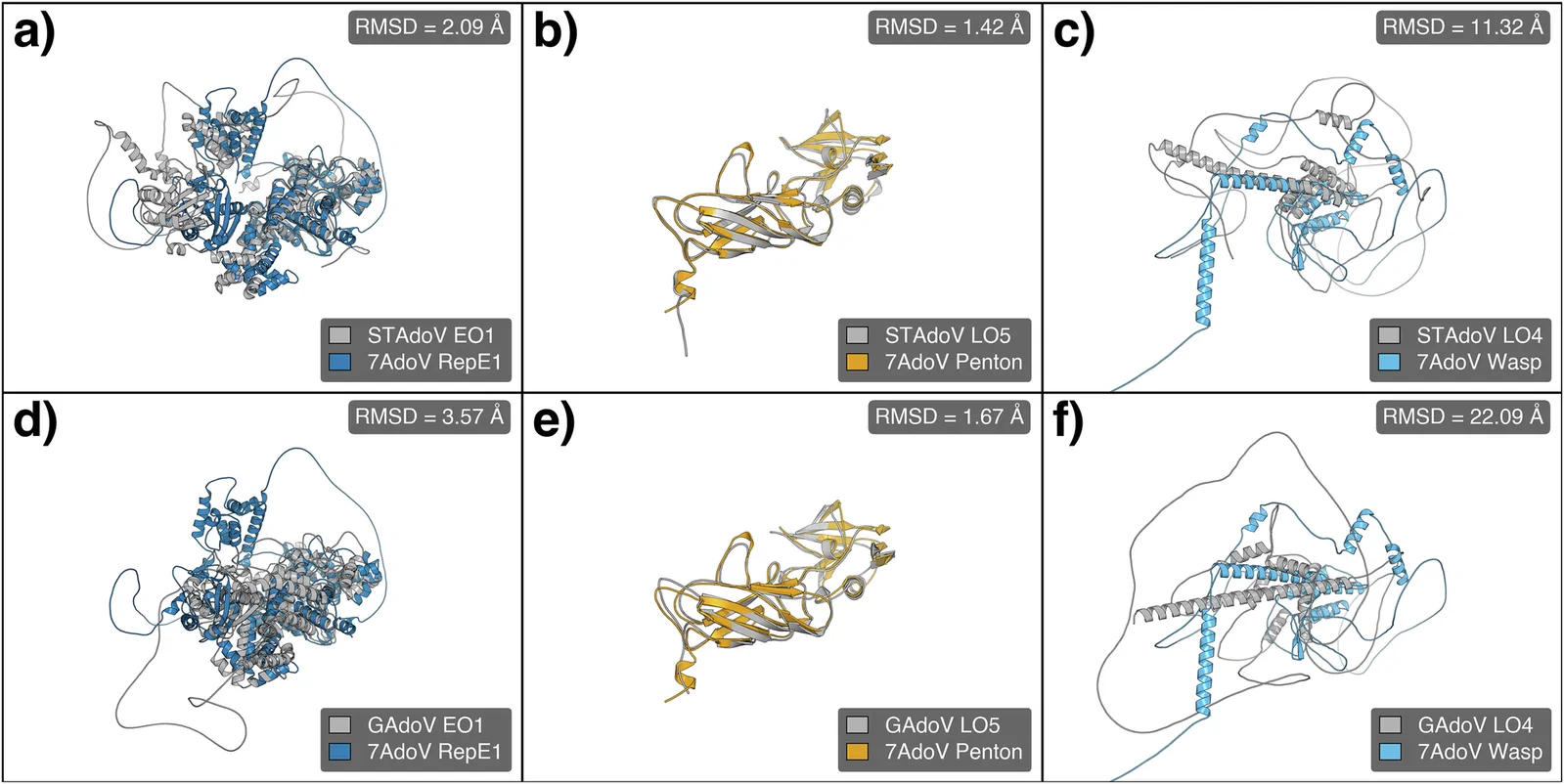
Structural comparisons of 7AdoV, STAdoV, and GAdoV proteins. AlphaFold-predicted structures for STAdoV (**a**–**c**) and GAdoV (**d**–**f**) proteins were aligned with their respective 7AdoV homologs RepE1 (**a**, **d**), Penton (**b**, **e**), and Wasp (**c**, **f**). Root mean square deviations (RMSD) are shown in Ångstroms for each pairwise comparison. 7AdoV protein structures are shown in color in fixed arrangements.

Structural homology searches of 7AdoV protein sequences with HHpred^29,30^ and AlphaFold^31^-predicted protein structures with Foldseek^32^ provided insight into the genomic organization and annotation of the circular adomavirus genome (Table 2, Fig. 2). SET, Penton, Macc, Hexon, Adenain, Prim, and RepE1 showed high structural homology to proteins with known function as well as adomavirus proteins; Col, Wasp, Cah, and Oncoid showed weaker structural homology to known proteins and were named based on synteny, weak HHpred hits, general structural features, or linear motifs, respectively (Table 2, Supplementary Fig. 1, Supplementary Data 1, 2). AlphaFold-predicted structures of the 7AdoV proteins RepE1, Penton, and Wasp were compared to the predicted structures of homologous STAdoV and GAdoV proteins (Table 2, Fig. 3). Root mean square deviation (RMSD) was determined between these proteins using the super alignment in PyMOL. Penton homologs from STAdoV (Fig. 3b) and GAdoV (Fig. 3e) showed the lowest RMSD to 7AdoV Penton among the compared proteins. The Penton homolog from JEECV showed a similar RMSD to 7AdoV Penton (Supplementary Fig. 2). STAdoV RepE1 (Fig. 3a) displayed a slightly lower RMSD to 7AdoV RepE1 than GAdoV RepE1 (Fig. 3d). Both Wasp homologs displayed high RMSD values (Fig. 3c, f).

**Table 2:** Predicted structural descriptions of the 11 7AdoV proteins are paired with their prior JEECV, GAdoV, and STAdoV homolog nomenclature

Description	7AdoV	JEECV	GAdoV	STAdoV	HHpred	AlphaFold-Foldseek
homolog of histone-lysine N-methyltransferase Su(var)3-9 Enhancer-of-Zeste and Trithorax (SET) proteins	SET*	–	SET*	SET*	9EH2_l, *P* = 99.77%	A0A6F9EYM8, E = 3.107E−14
ORF syntenic to adomavirus collagen-like LO1 genes	Col	LO1	–	–	PF07150.18, *P* = 23.62%	A0A6M3T0N8, E = 2.728
remote C-terminal similarity to Wiskott-Aldrich syndrome protein-homology domain 2 (WH2) containing proteins	Wasp	LO2,3	LO4	LO4	cd22080, *P* = 66.49%	A0A7R8MJK0, E = 1.005
candidate homolog of adomavirus capsid-surface alpha helix-rich proteins	Cah	LO4	–	–	2JDI_I, *P* = 28.69%	AF-A0A941JV34-F1, E = 0.6851
similarity to the pentameric single-jellyroll vertex capsomer of adenoviruses	Penton	LO5	LO5	LO5	Q5UQF3, *P* = 99.95%	A0A2S1MK20, E = 5.891E−30
candidate membrane-active capsid core protein with similarity to adenovirus protein X	Macc	LO6	LO6	LO6	Q8JN69, *P* = 89.60%	A0A2S1MK17, E = 1.657E−13
structural similarity to the double-jellyroll hexon proteins of megaviruses	Hexon	LO7	LO7	LO7	PF21738.2, *P* = 96.37%	A0A2U9Q1M3, E = 5.777E−59
adenovirus-like virion maturation protease	Adenain	LO8	LO8	LO8	P68985, *P* = 99.88%	A0A161JDF9, E = 4.431E−18
large protein with N-terminal similarity to the small catalytic subunit of archaeal-eukaryotic primases	Prim	EO2,3,4*	EO2-4	EO3	6RB4_A, *P* = 99.59%	A0A6F9FAG1, E = 1.411E−20
short cysteine-rich protein with linear motifs reminiscent of viral oncogenes^33^	Oncoid*	–	–	–	PF23630.2, *P* = 62.41%	Q83439, E = 4.25
papillomavirus-like replicative superfamily 3 helicase	RepE1*	EO1*	EO1*	EO1*	P26543, *P* = 100%	A0A2S1MK29, E = 3.773E−77

Best HHpred probabilities and Foldseek E-values are found for each 7AdoV protein. HHpred predictions are reported with the accession for the top probability alignment from the NCBI Conserved Domain, PDB, Pfam, or UniProtSwissProt-viral70 database. Foldseek predictions are reported with the accession for the lowest E-value alignment for the AlphaFold-predicted structure to the AlphaFold, PDB, BFVD, MGnify, CATH50, GMGCL, or BFMD database. ORFs on the same strand as the replicase, the early strand, are denoted with an asterisk (*). Gene nomenclature follows the conventions presented in Buck et al.^22^.

### Assessment of 7AdoV gene expression and genome content

**Fig. 4:**
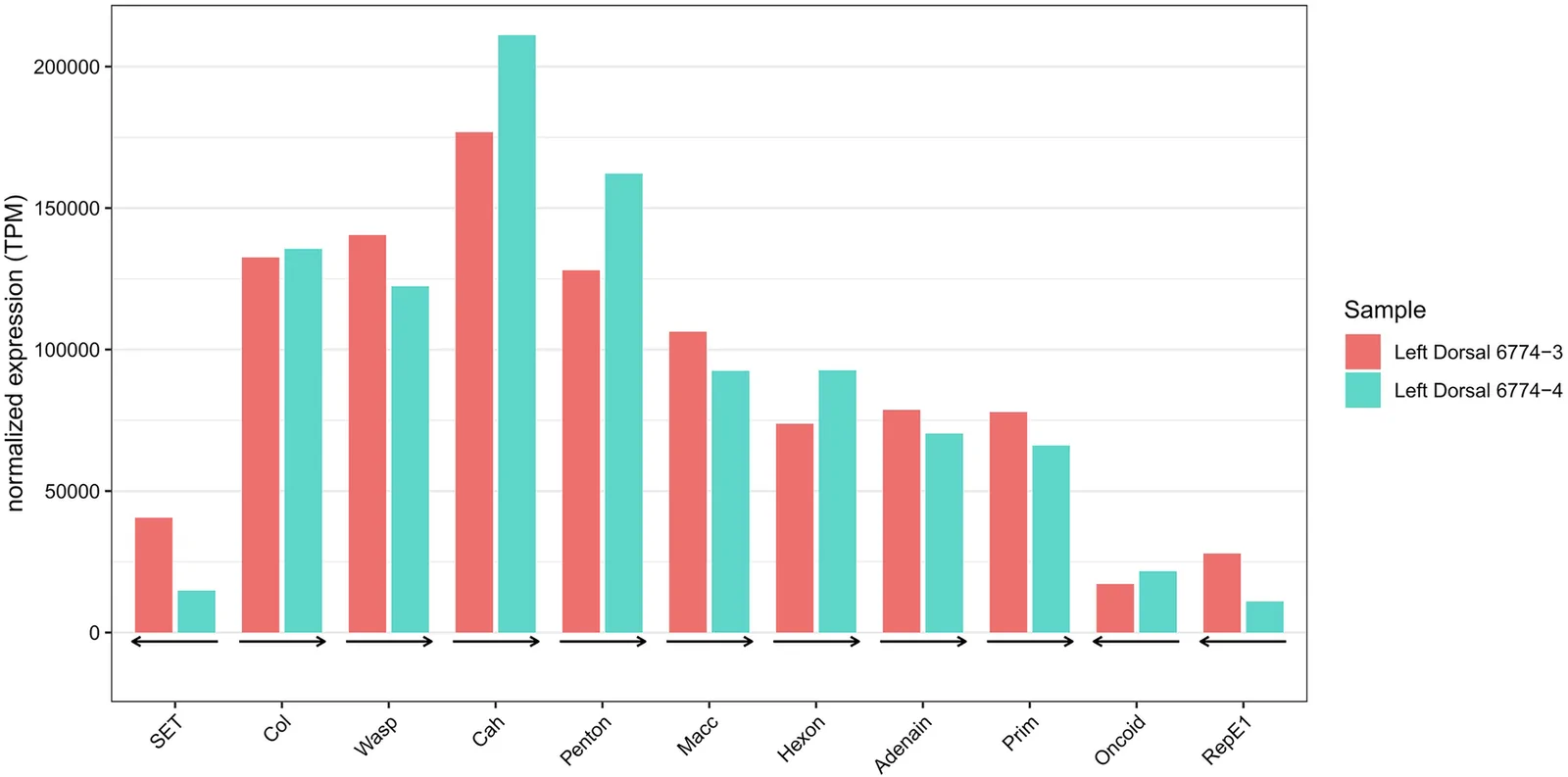
Expression of 7AdoV viral genes in lesioned tissue. The normalized expression of viral genes is shown for lesioned tissues from the broadnose sevengill shark. Genes are arranged in genomic order of open reading frames, with arrows indicating the coding strand. The height of the bars represents the normalized expression of each gene in transcripts per million (TPM).

The transcriptional profile of 7AdoV was generated for each lesioned sample by mapping RNA reads and normalizing to transcripts per million mapping (TPM) for each genomic feature (Fig. 4). Normalized viral gene expression was similar in both samplings of lesioned tissue, with late strand genes having higher expression than early strand genes. Tandem repeat detection revealed several tandem repeat regions within the Prim coding sequence. Notably, three hexameric repeats were detected, which correspond to repeated amino acid sequences of AP, AG, and RE. These amino acid repeating pairs appear 37, 33, and 14 times, respectively, in the full amino acid sequence (accession XHO08303).

### Phylogenetic analysis places 7AdoV amongst RepE1 adomaviruses

**Fig. 5:**
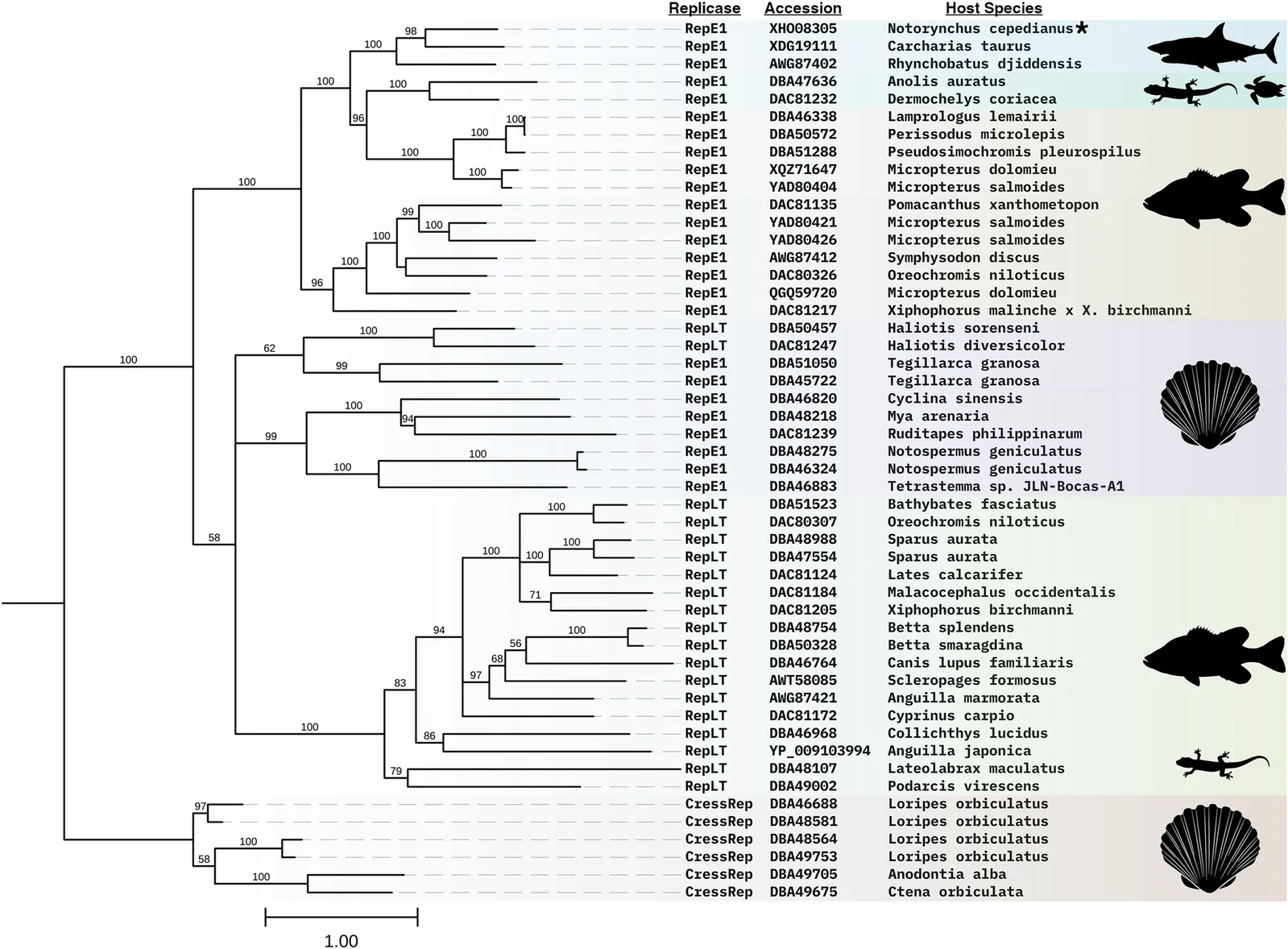
Maximum likelihood tree placing 7AdoV RepE1 amongst adomavirus replicases. Phylogenetic analysis was performed on the RepE1 protein of 7AdoV to place it amongst the replicases of other known adomaviruses. The resulting consensus tree is shown with bootstrap values indicated on all branches with support >55% and a scale bar indicating substitutions per site. 7AdoV is indicated with an asterisk (*). Highlight colors are used to group adomavirus subclades and icons are used to represent general host taxonomy. From top to bottom, elasmobranch RepE1 adomaviruses are in light blue, reptile RepE1 adomaviruses are in light teal, and teleost RepE1 adomaviruses are in light beige. Invertebrate RepE1 and RepLT adomaviruses are highlighted in lavender. Teleost and reptile RepLT adomaviruses are highlighted in light green and invertebrate CressRep adomaviruses are highlighted in light peach.

Phylogenetic analysis of 7AdoV RepE1 amongst adomavirus replicase proteins placed it in a clade with the elasmobranch, reptile, and bony fish RepE1 adomaviruses (Fig. 5, Supplementary Data 3a). This subclade lacked clam and ribbon worm RepE1s, which were assigned to other subclades that included abalone RepLTs. Elasmobranch adomavirus RepE1s formed a distinct subclade, with STAdoV RepE1 sharing the most recent common ancestor with 7AdoV RepE1 (Fig. 5). Similarly, the bony fish and reptile RepLT sequences formed their own clade, while CressRep sequences were assigned to their own clade (Fig. 5).

### RNAscope in situ hybridization localizes virus to lesioned tissue

**Fig. 6:**
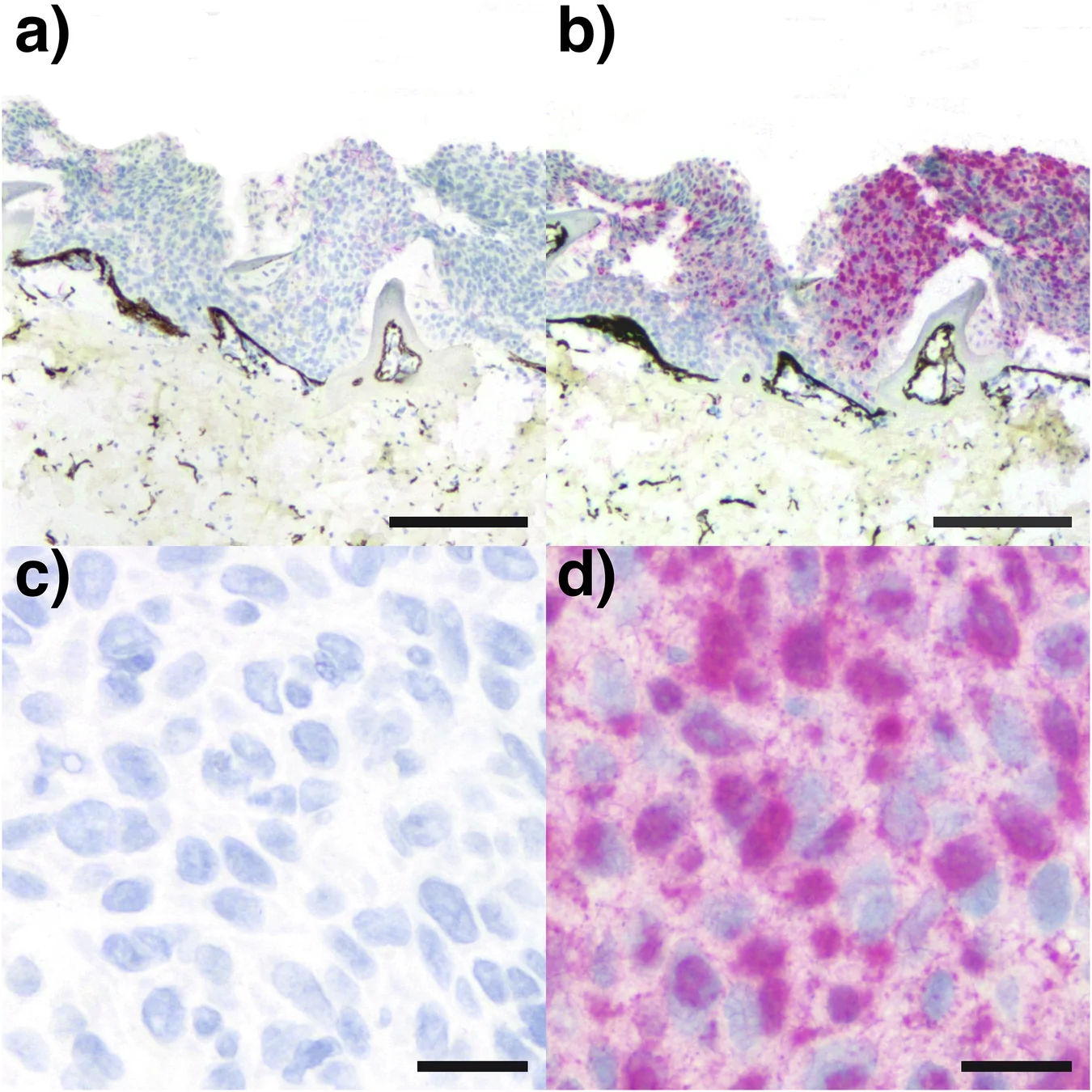
Singleplex chromogenic RNAscope in situ hybridization performed on lesioned skin. Low (**a**, **b**) and high (**c**, **d**) magnification images of histologic sections of lesioned skin with epidermal hyperplasia. No hybridization signal is apparent in histologic sections to which the negative control dapB probe was applied (**a**, **c**). There is variably intense red hybridization signal across the hyperplastic epidermis to which the 7AdoV Wasp probe was applied (**b**), with clumped red nuclear labeling accompanied by punctate red peri-nuclear labeling in epithelial cells (**d**). Hematoxylin counterstain. Scale bar = 200 µm (**a**, **b**) or 20 µm (**c**, **d**).

Singleplex chromogenic RNAscope in situ hybridization was used to determine the anatomic distribution of adomavirus transcripts in histological sections of sevengill shark skin. Hybridization signal from the 7AdoV Wasp probe was restricted to nuclei and perinuclear cytoplasm of epithelial cells in the hyperplastic epidermis (Fig. 6, Supplementary Fig. 3). Transmission electron microscopic (TEM) examination of skin specimens with epidermal hyperplasia from the same sampling as that used for RNAscope in situ hybridization did not reveal virions in spinous epithelial cells.

## Discussion

7AdoV is one of a short, but growing list of adomaviruses to be detected in elasmobranchs^13,14^. Most recently, a RepE1 adomavirus was detected and associated with proliferative skin lesions in a sand tiger shark (STAdoV)^14^. Discovery of a novel adomavirus in an aquatic organism is not unexpected, as host range has been shown to extend across farmed^19^, displayed^18^, and wild^34,35^ teleosts and elasmobranchs maintained in aquariums^13,14^. The source and timing of the 7AdoV infection in this broadnose sevengill shark is unknown. Elasmobranchs in captivity have shown development of progressive skin lesions following stress events^6^, and it has been postulated that the stress associated with capture, handling, and relocation has the potential to induce immunosuppression and allow for opportunistic infection^6^. Aquarium settings can provide artificial interactions of hosts otherwise less likely to occur in the wild, allowing for previously or newly acquired infections to manifest^6^. This adomavirus, as well as the two previous elasmobranch adomaviruses in the guitarfish and sand tiger shark, were identified after wild-caught animals were transported to aquarium settings^13,14^. This association may also be correlative, as study of disease prevalence in wild fish populations is limited by practical barriers^6,36^.

Consequences of adomavirus infections vary widely from significant commercial fishing losses associated with JEECV^19^ to self-limiting proliferative lesions in individuals associated with GAdoV^13^, STAdoV^14^, and 7AdoV. To date, many adomavirus sequences have been identified using database mining; however, their further characterization and association with disease are undetermined^18,22^. Adomaviruses have been associated with clinical presentations of lethargy and inflamed skin lesions in a discus cichlid^18^, hyperpigmented melanistic lesions in both smallmouth and largemouth bass (blotchy bass syndrome)^35^, and extensive hemorrhaging in the gills of Japanese^37^, marbled^20^, and American^38^ eels. Adomavirus infections have also been detected in clinically healthy animals (arowana adomavirus)^18^. Information regarding adomavirus transmission and progression is limited. STAdoV was shown to infect conspecifics^14^. Separate lesions associated with smallmouth bass adomaviruses have been shown to expand and recede on the same individual^39^, and some individuals have demonstrated resolution of skin lesions in other adomaviruses, e.g. 7AdoV, GAdoV, and STAdoV^13,14^. Different adomavirus disease presentations in individual hosts have been hypothesized to be dependent on host conditions^38^, environmental stressors^39^, or even coinfections with other viruses^40^.

In the current case study, a broadnose sevengill shark developed a series of proliferative epidermal lesions that persisted over the course of several months. Biopsy of the lesioned skin revealed hyperplasia of spinous epithelial cells of the epidermis. Metagenomic sequencing identified a novel adomavirus with distant nucleotide similarity to the conserved RepE1 gene in GAdoV. After circularizing the candidate virus genome, protein-space alignments with BLASTx and BLASTp detected adomavirus homologs for most of the remaining virus features. HHpred remote homology scans and Foldseek searches of AlphaFold-predicted structures further supported assignment of 7AdoV proteins descriptive names. These descriptive names are not intended to imply the structure or function of these proteins but instead attempt to facilitate future homolog recognition^22^. Replication-associated SET, RepE1, and Oncoid proteins are encoded on the inferred early strand, while adenovirus-like structural and morphogenetic proteins Cah, Penton, Macc, Hexon, and Adenain are encoded on the late strand with Col, Wasp, and Prim (Fig. 2, Table 2). One further benefit of this descriptive nomenclature is that strandedness is not implied. Prim, syntenic to EO2-4 ORFs in JEECV, is located and expressed as a late strand gene in 7AdoV (Fig. 4), similar to the syntenic genes in the blotchy bass syndrome-associated adomaviruses^35^.

Phylogenetically, 7AdoV resolved among other elasmobranch RepE1 adomaviruses most closely to STAdoV (Fig. 5). Vertebrate RepE1 adomaviruses formed a well-supported single clade with subclades observed for elasmobranch, reptile, and teleost hosts. Similarly, vertebrate RepLT sequences formed a clade consisting primarily of teleost hosts. A RepLT adomavirus from a dog was noted in this clade; however, as this sequence was recovered from a saliva metagenome and resolved closely with bony fish adomaviruses, it likely does not demonstrate a definitive host expansion to mammals. When considering the broader tree topology, neither RepE1 nor RepLT adomaviruses resolved as monophyletic groups across all hosts; instead, separate clades were observed for invertebrate adomaviruses, one containing only RepE1 adomaviruses, and one containing both RepE1 and RepLT adomaviruses. CressRep adomaviruses, which have been identified exclusively in invertebrate hosts, formed a monophyletic clade after re-rooting. This expanded phylogenetic analysis is consistent with previous studies^35^, while broadening host representation across both vertebrate and invertebrate taxa.

RNAscope in situ hybridization localized virus transcripts of the Wasp gene to regions of hyperplastic epidermis, providing a histological and spatial context for the results of the bulk metagenomic analysis. Attempts to detect 7AdoV virions in lesioned skin using transmission electron microscopy were unsuccessful. In comparison, although virion arrays were observed in TEM imaging of keratinized skin spicules caused by trichodysplasia spinulosa polyomavirus (HPyV8)^41^, virions have never, to the authors’ knowledge, been directly visualized by TEM in skin infected with other common human polyomaviruses (HPyVs 5, 7, and 10). Failure to detect viral particles was similarly noted in STAdoV infection, which could have been a consequence the timing of sampling, i.e. after productive viral replication had ceased^14^.

The hyperplasia, regression, and resolution of the skin lesions associated with 7AdoV in this sevengill shark extend the trend noted by Powell et al.^14^ and Iwanowicz et al.^35^, i.e. that lesions associated with RepE1 adomaviruses have been proliferative involving epithelium of the skin. This is in contrast to the lesions identified in RepLT adomavirus infections, which have been necrotizing and hemorrhagic, involving endothelium of the gills and internal organs (often liver and kidney)^20,37,38^. While these current trends in disease presentation exist, it is possible our understanding of them may change as further adomavirus genomes and presentations are discovered. This study presents the first picture of RepE1 adomavirus gene expression in an elasmobranch adomavirus (Fig. 4). In 7AdoV, gene expression of the late structural gene set was markedly higher than the early replicative gene set indicating a late stage of viral infection at the time of sampling^42^. This is correlative with both the pathological and clinical presentation of the infection and is consistent with the expression of viral genes in late-stage infections of the blotchy bass syndrome-associated adomaviruses^35^. This is also in contrast to the genomic coverage of 7AdoV (Fig. 2); although repeat regions in 7AdoV show some variable coverage differences, genomic coverage is relatively consistent across the rest of the genome. Transcriptomic coverage is shown instead to vary relative to coding strand.

Differences observed in RepE1 and RepLT adomavirus presentations may lie in how the viruses regulate gene transcription and thereby affect the host cell cycle. In many polyomaviruses and papillomaviruses, an LXCXE or LXXLFD oncogenic domain binds to retinoblastoma (Rb) and other pocket proteins to disrupt cell cycle regulation^43^. While some RepLT adomaviruses potentially encode this motif^18^, 7AdoV, as well as GAdoV and STAdoV, instead encode an N-terminal RepE1 motif, VRTLFD, that might bind the minor groove of pocket proteins (Eukaryotic Linear Motif DOC_CYCLIN_RxL_1)^44,45^. Prim also contains several short hexanucleotide tandem repeats within its coding sequence, reminiscent of the rearrangements of regulatory regions of polyomavirus genomes found in pathogenic conditions^46^. In these polyomavirus non-coding control regions, larger (>20 bp) variable number tandem repeats have been shown to act as early viral transcription enhancers^47,48^. Although short (<10 bp) tandem repeats are generally enriched in viral genomes, hexameric repeats are less common in some viral phyla, potentially due to compact genomes and overlapping reading frames^49^. When they do appear, hexameric repeats are more likely to be found in coding regions than other short tandem repeats besides tri- and nonamers^49^. Zhao et al. showed that hexanucleotide repeats appear in only ~2.6% of 10–30 kilobase virus genomes^50^. Interestingly, the double-stranded DNA virus in their study closest in size to 7AdoV with a hexamer repeat was human adenovirus C, where the repeat is found in a DNA encapsidation gene (accession YP_001551767)^50^. A tandem hexameric repeat is also found in the coding sequence of EO3 in a smallmouth bass adomavirus (accession MN631022)^34^, syntenic to 7AdoV Prim. These rare coding repeats may support the suggestion in Buck et al. that Prim is derived from ancestral eukaryotic viruses and horizontally transferred to adomaviruses, but not further to papillomaviruses or polyomaviruses^22^.

The structural conservation and potential functions of adomavirus proteins were hypothesized using a predictive and comparative approach. The predicted Penton and Hexon proteins showed single- and double-jellyroll structures, respectively (Supplementary Fig. 1g, d), key features of adenovirus capsids^51^. Wasp and Prim were named based on limited domain similarity to known proteins (Table 2). Despite the high domain identity of Prim, this composed a small fraction of the total ORF. Structural comparisons of homologs between 7AdoV, STAdoV, and GAdoV showed higher conservation of adenovirus-like capsid genes compared to replicase genes (Fig. 3). RMSDs less than 2 Å, a typical threshold for highly similar proteins^52^, were found for both STAdoV and GAdoV homologs aligned to 7AdoV Penton (Fig. 3b, e). The RepE1 protein of 7AdoV showed higher structural similarity to that of STAdoV compared to that of GAdoV (Fig. 3a, d), which may indicate that replicase protein structures are comparatively less conserved than adomavirus capsid protein structures. This is further supported by the alignment between 7AdoV Penton and the homologous protein in JEECV, which showed an RMSD of 1.68 Å (Supplementary Fig. 2). The protein structure of 7AdoV Wasp showed little similarity to homologs from STAdoV and GAdoV (Fig. 3c, f). A similar reduction in identity of proteins upstream of the Cah locus was previously noted in comparisons of the blotchy bass adomaviruses^35^.

Using a combination of pathological, molecular, and transcriptomic evidence, it was possible to identify and characterize a novel RepE1 adomavirus from a broadnose sevengill shark with proliferative skin lesions. The molecular virology workflow that was used allowed for detection of the virus from a limited sample set using a metagenomic approach and further allowed for virus and host probe design for RNAscope in situ hybridization, which localized virus transcripts to sites of epidermal hyperplasia. This workflow resulted in the characterization of an 18,834 bp adomavirus, the broadnose sevengill shark adomavirus (7AdoV). The genome of this adomavirus encoded replication-associated genes and adenovirus-like morphogenetic genes. Phylogenetic analyses placed this adomavirus among other elasmobranch adomaviruses, all of which encode E1-like replicase proteins (RepE1s). Structural comparisons of replicase and capsid proteins of 7AdoV to those of adomaviruses from other elasmobranchs displayed high degrees of structural similarity. This workflow offers a potential mechanism for virus profiling that bypasses certain limitations of novel virus informatics and understudied host organisms.

## Methods

### Ethics statement

All tissue samples were collected from a live broadnose sevengill shark (*Notorynchus cepedianus*) for diagnostic purposes by a veterinarian licensed by the State of California and employed by the Monterey Bay Aquarium (California, USA) in accordance with standards and policies for the humane treatment of all animals under institutional care. The Monterey Bay Aquarium is an institution accredited by the Association of Zoos & Aquariums (AZA-Accredited Members; https://www.aza.org/inst-status). Accreditation ensures that members meet the AZA standards for animal welfare, care, and management (2026 AZA Accreditation Standards and Related Policies; https://assets.speakcdn.com/assets/2332/accred_standards.pdf). This broadnose sevengill shark was collected in accordance with the California Department of Fish & Wildlife (Permits SC-2026 and S-190810004-21007-001) for the conservation of the species in California.

### Disease presentation and sample collection

A juvenile (weight: 10.8 kg, straight length: 137 cm) male broadnose sevengill shark (*Notorynchus cepedianus*) was captured in San Francisco Bay on Sept 1, 2021. The shark was introduced and managed under human care in a community tank of approximately 315,000 gallons (1,192,405 liters) of ambient sea water. During a routine examination on May 17, 2022, a series of lesions were noted on the skin of the shark and skin scraping was performed. Temporary non-chemical anesthetic restraint employing a combination of tonic immobility^53^ and hyperoxygenation (180–200 ppm) of the water in a rectangular shark restraint box was employed to collect two 6 mm Baker’s biopsy punch samples for histologic evaluation. Based on the results of the initial biopsies, a decision was made to repeat the immobilization and biopsy to collect additional samples for more advanced evaluations. Six-millimeter biopsy specimens were collected from lesioned areas, left dorsal and caudal left dorsal, and non-lesioned areas, left ventral and right ventral. Each biopsy sample was bisected with a new single-edge razor blade. One half was preserved in 10% neutral buffered formalin, and the other half in molecular transport medium (Primestore MTM, Longhorn Vaccines and Diagnostics, Bethesda, MD). Two blood samples were collected at the time of the second biopsy procedure. After collection, all samples were shipped to the New Hampshire Veterinary Diagnostic Laboratory (Durham, NH). Upon receipt, one sample of coagulated blood was centrifuged, and buffy coat was isolated and transferred to molecular transport medium. Skin and blood samples were submitted to the Hubbard Center for Genome Studies (University of New Hampshire, Durham, NH) for transcriptomic and genomic analyses. Formalin-fixed biopsy samples of skin were decalcified by overnight immersion in a solution of 0.5 M ethylenediamine tetra-acetic acid (EDTA), then processed routinely for paraffin embedment and histological sectioning. Histological sections were prepared at 4 µm thickness, stained using hematoxylin and eosin, then examined by bright field microscopy.

### Whole-genome and whole-transcriptome sequencing

The six samples arrived at the Hubbard Center for Genome Studies as unfixed skin tissue and coagulated blood. Total nucleic acid (DNA and RNA) was extracted using the MagMAX Microbiome Ultra Nucleic Acid Isolation Kit (ThermoFisher). DNA libraries were prepared using the KAPA HyperPlus Kit (Roche). RNA libraries were prepared using the MICROBExpress™ Bacterial mRNA Enrichment Kit (ThermoFisher) followed by KAPA RNA HyperPrep Kit (Roche). Samples were sequenced at the Hubbard Center for Genome Studies on a NovaSeq 6000 (Illumina) using paired end, version 1.5 chemistry on an SP, patterned flow cell. Forward and reverse read lengths were 250 bp and indexing reads were dual 8-mers. The data were demultiplexed using Illumina bcl2fastq v2.20.0.422 (Illumina) and checked for quality using FastQC v0.11.5.k (https://www.bioinformatics.babraham.ac.uk/projects/fastqc/).

### Read assessment and per-sample assembly

Raw forward and reverse read FASTQ files were assessed using Trimmomatic PE v0.39^54^ with a 4-base wide sliding window removing bases with an average quality below 10 and any adapter sequences. Trimmed DNA reads were assembled using SPAdes v3.13.0^55^ run with default settings and supplying unpaired reads, resulting in single DNA assemblies for each sample. These DNA assemblies were assessed for quality using QUAST v5.0.2^56,57^. The mitochondrial DNA of *N. cepedianus* was downloaded from GenBank (accession AB560489) and was queried against each individual assembly using blastn v2.11.0+^58^ to confirm the taxonomic identity of the host organism. Trimmed RNA reads from each sample were concatenated into single forward and reverse FASTQ files and assembled using Trinity v2.1.1^59,60^ with default settings.

### Combined host-depleted DNA assembly

The genome assembly of the small-spotted catshark (*Scyliorhinus canicula*) was downloaded from National Center for Biotechnology Information (NCBI) RefSeq on February 16, 2023 (sScyCan1.1). *S. canicula* represented the closest relative to *N. cepedianus* with a RefSeq assembly at the time of study, sharing taxonomy at the infraclass *Selachii*^61^. The small-spotted catshark genome was indexed, and trimmed DNA reads from each sample were individually mapped to the genome using BWA-MEM v0.7.17-r1188^62^. The sequence alignment/map (SAM) files were converted to binary alignment map (BAM) and indexed using SAMtools v1.10^63^. Read pairs with either mate not mapping to the small-spotted catshark genome were isolated into FASTQ files. These host-depleted reads from each sample were combined into single forward and reverse FASTQ files and assembled using SPAdes without supplying unpaired reads. Coverage metrics and mapping files were generated for the assembly by mapping the host-depleted reads against the assembled contigs using BWA-MEM and SAMtools as above.

### Taxonomic assessment of DNA contigs

The combined host-depleted assembly was characterized using MegaBLAST run against the NCBI nucleotide (nt) database^64^ downloaded locally on November 6, 2021. The blastn program was run with the following parameters: “-task megablast -db <nt>-culling_limit 5 -evalue 1e-5 -outfmt ‘6 qseqid staxids bitscore’“. Taxonomy tables were created using Blobtools v1.1.1^65^ with the following commands “blobtools create -i <host_depleted_contigs>-b <host_depleted_bam> –t <megablast_hits>-o <out_prefix>; blobtools view -i <out_prefix_blobDB.json>-r all”. By default, Blobtools uses the “bestsum” and “bestsumorder” to assess the best taxonomic ranking for a contig; if no hits existed in the MegaBLAST file, contigs were taxonomically labeled as “no-hit”. All “no-hit” contigs were isolated into their own FASTA file and a second round of BLAST against the nt database was run, this time using the less stringent “-task blastn” without culling limits or e-value thresholds. Manual searches were used on the BLASTn file to identify virus hits.

### Genomic confirmation of the adomavirus

An 18,859 bp “no-hit” contig from the combined host-depleted DNA assembly was identified and characterized by several BLASTn hits to nt adomaviruses. This contig was further confirmed from a BLASTx query against nt revealing eight unique adomavirus hits. Viral screening of the combined host-depleted assembly was also completed using CenoteTaker3 v3.3.2 with the flags “-p T --lin_minimum_hallmark_genes 2” as suggested for microbial metagenomes^25^ followed by CheckV for quality and completeness^26^. The virus contig was annotated using PROKKA v1.14.6^66^ and resulting hypothetical protein coding sequences were characterized using the best hit from BLASTp run against the non-redundant protein database, confirming a near-complete adomavirus genome.

### Genome polishing and refined adomavirus annotation

To improve virus resolution, a modified version of the Circlator v1.5.5^67^ workflow was followed. The combined host-depleted DNA reads were mapped back to the virus contig, and mapping reads were isolated into their own paired-end FASTQ files and reassembled using SPAdes as above. Small and completely contained contigs were removed using “circlator clean”, resulting in a single 17,757 bp virus contig. The start position of the sequence was adjusted to just before the SET gene (annotated from PROKKA) using “circlator fixstart --genes_fa <SET.fasta>“ and the sequence was reverse complemented to follow prior adomavirus annotation conventions^13^.

Read mapping of the host-depleted DNA reads against this draft genome revealed an unusually highly covered section of the adomavirus genome in the Prim gene corresponding to a tandem repeat annotated by Tandem Repeats Finder^68^. PCR primers (5’–GCCAGGTTTGACAAGTCGAA–3’ and 5’–GATATTCCGGCTGCATCGTC–3’) were designed using Primer3 v4.1.0^69^ to flank the detected repeat region and remain within the Prim gene. Extracted DNA from lesioned left dorsal tissue (6774-4) was subjected to long range PCR with the LongAmp™ Taq PCR Kit (New England Biolabs) followed by standard AMPure XP cleanup (Beckman). Resulting products were ligated using the Ligation Sequencing Kit V14 (SQK-LSK114, Oxford Nanopore Technologies) and a 12.6 ng library was loaded onto an R10 MinION flow cell and ran for 72 h on the GridION with MinKNOW basecalling (Oxford Nanopore Technologies).

Long reads spanning the tandem repeat region were used to update the sequence of the draft genome. This updated genome was then subjected to two rounds of short-read polishing using the Polypolish v0.6.1^70,71^ workflow with the host-depleted DNA reads. After two rounds of polishing, no further changes were made, and the resulting sequence was considered the complete 7AdoV genome. Refined ORF annotations of the genome were done using PROKKA^66^, ORFfinder^72^, and MacVector 18^73^. Motif detection was performed on the 7AdoV genome using the Eukaryotic Linear Motif resource^44^.

Amino acid sequences of all detected ORFs were input into AlphaFold 2.3.2^31^ to generate protein structural predictions. All structures were visualized using PyMOL (Schrödinger, Inc.). These structures were queried against the AlphaFold, PDB, BFVD, MGnify, CATH50, GMGCL, and BFMD databases using Foldseek^32^ and annotated based on E-values. Further annotations were derived from the top probability hit from scans of ORFs against the NCBI Conserved Domain, PDB, Pfam, and UniProtSwissProt-viral70 databases using web-based HHpred^29,30^. STAdoV and GAdoV proteins homologous to 7AdoV RepE1, Penton, and Wasp, as well as the JEECV homolog to 7AdoV Penton, were similarly input into AlphaFold to predict their structures (accessions XDG19111, XDG19104, XDG19107, YP_009552707, YP_009552702, YP_009552701, YP_009103985). The predicted structures were aligned to their 7AdoV homolog with the “super” command to calculate RMSD and visualized using PyMOL (Schrödinger, Inc.).

### Assessment of 7AdoV genome content and gene expression

Combined host-depleted DNA reads were mapped to the masked adomavirus genome to generate genomic coverage metrics and median coverage from SAMtools depth was reported. Combined trimmed RNA reads were mapped to the adomavirus genome to generate transcriptomic coverage data. The 7AdoV genome was diagrammed using pyCirclize (https://github.com/moshi4/pyCirclize) to show ORF organization, genomic and transcriptomic coverage, and GC-content. Prior to generating per-sample gene expression metrics with short reads, simple repeats in the 7AdoV genome were masked with RepeatMasker v4.0.7 (https://www.repeatmasker.org/) using default settings. Trimmed RNA reads from each sample were mapped to the masked adomavirus genome with BWA-MEM, after which featureCounts v2.1.1^74^ was used with parameters “-B -p -O --countReadPairs -t gene” to count total transcript fragments per gene feature. These counts were normalized by feature length and converted to transcripts per million (TPM) following Zhao et al.^75^.

### Phylogenetic analysis of adomavirus replicase protein sequences

Phylogenetic analyses were run on the 7AdoV RepE1 amino acid sequence to place the novel adomavirus among the Adomaviridae. Adomavirus RepE1, RepLT, and CressRep sequences were curated from NCBI Viruses. In total, 50 adomavirus replicase protein sequences were included in the analysis. GenBank accessions for these adomavirus proteins are found in Supplementary Data 3b. Alignment was performed using Clustal Omega^76^ with the Clustal output and default settings. The alignment length corresponded to the length of the longest replicase, the RepLT of Adomavirus podarcis19746 (accession DBA49002), at 1668 amino acids. Bootstrapped maximum likelihood phylogenetic analyses were performed using IQ-Tree v1.6.12^77,78^, run with the parameters “-st AA -bb 1000 -minsup 0.5”. The VT + F + R4 amino acid substitution model^79^ was selected as the best-fit model by ModelFinder^80^. The consensus phylogenetic tree was visualized using TreeViewer v2.1.0^81^ and rooted using CressRep sequences as the outgroup based on prior conventions^35^.

### Probe design and spatial localization of the adomavirus by RNAscope

The Wasp gene transcript was selected for in situ probe design due to it having the highest average TPM among genes sufficient in length for RNAscope probe design. A positive control *peptidylprolyl isomerase B* (*PPIB*) gene transcript was also extracted from the host combined transcriptome using MegaBLAST querying of a great-white shark *PPIB* (accession XM_041178444). The transcript with the largest hit to the reference *PPIB* was fed into TransDecoder v3.0.1 (https://github.com/TransDecoder/TransDecoder) to detect the open reading frame of the *N. cepedianus PPIB*. Virus transcripts were subtracted from the combined transcriptome to generate a background and detect probe cross-reactivity. Sequences of the Wasp gene transcript and the sevengill *PPIB* gene transcript were submitted to ACDBio (Newark, CA) for probe design. The 7AdoV Wasp probe targeted the 2756–3691 bp region of the 7AdoV genome, and BLASTn searches of the probe against nt revealed no nucleotide sequence identities.

The 7AdoV Wasp probe (LS, V-AdoV-L04-C1, Cat No. 1233728-C1) in conjunction with the *PPIB* probe (LS, Nc-PPIB-O1, Cat No. 1234238-C1) and a *dihydrodipicolinate reductase* (*dapB*) negative control probe (LS, Cat No. 312038) was used in singleplex chromogenic in situ hybridization reactions performed on the Leica Biosystems BOND RX automated IHC/ISH slide staining platform (Leica Biosystems, Deer Park, IL) by the Pathology Shared Resource in the Section for Clinical Genomics and Advanced Technology, Department of Pathology and Laboratory Medicine (Dartmouth Health System, Lebanon, NH). Histological sections of formalin-fixed, paraffin-embedded (FFPE) lesioned and non-lesioned shark skin were tested using a modified protocol to that of Anderson et al.^82^, with histological sections from each sample subjected to hybridization using a *dapB* negative control, sevengill shark *PPIB* positive control, and Wasp test probe. Histological sections prepared at 4 µm thickness were baked at 60 °C for 30 min prior to being loaded to the Leica Biosystems BOND RX instrument. Sections were deparaffinized on the instrument, then underwent target retrieval using Leica BOND Epitope Retrieval Solution 2 (Cat No. AR9640, Leica Biosystems Inc. Buffalo Grove, IL) for 15 min at 88 °C, followed by protease incubation for 15 min at 40 °C. Probes were hybridized for 2 h at 42 °C, followed by RNAscope signal amplification and fast red chromogen detection using the RED RNAscope®2.5 LS (Leica Biosystems, Cat No. 322150) kit. Images were visualized using QuPath v0.4^83^ and PMA.start v2.1.1.2085 (Pathomation BV, Berchem, Belgium).

### Transmission electron microscopy of lesioned skin

A sample of FFPE lesioned skin, determined by histopathological assessment and RNAscope adomavirus probe labeling, was re-embedded in paraffin and sent to the Electron Microscopy Laboratory, Department of Pathology, University of Texas Medical Branch (Dr. Vsevolod Popov, Galveston, TX). Foci of interest determined on histological sections were excised from the paraffin block, deparaffinized in xylene, hydrated, fixed in a modified Karnovsky’s fixative—a mixture of 2% formaldehyde prepared from paraformaldehyde and 2% glutaraldehyde in 0.1 M cacodylate buffer pH 7.4—and then processed and embedded in epoxy resin as described previously^84,85^. 80-nm ultrathin sections were stained with lead citrate and examined in a JEOL-1400 transmission electron microscope (JEOL USA, Peabody, MA) at 80 kV equipped with a bottom-mounted CCD camera Orius SC2001 (Gatan, Pleasanton, CA).

## Supplementary information

Supplementary Information

Supplementary Data 1

Supplementary Data 2

Supplementary Data 3

## Data Availability

All relevant data are within the manuscript and its Supplementary Information files. The raw DNA and RNA reads used have been submitted to the SRA under BioProject PRJNA1157825, and the complete 7AdoV genome is deposited to GenBank under accession PQ316599.
