# Supplementary material for: A novel adomavirus from proliferative skin lesions of a broadnose sevengill shark (Notorynchus cepedianus)
Source: Npj Viruses. 2026 Jul 2;4:51. doi: 10.1038/s44298-026-00210-8 (PMC13696692; doi:10.1038/s44298-026-00210-8)
Supplement: Supplementary file 1 [file 44298_2026_210_MOESM1_ESM.pdf]

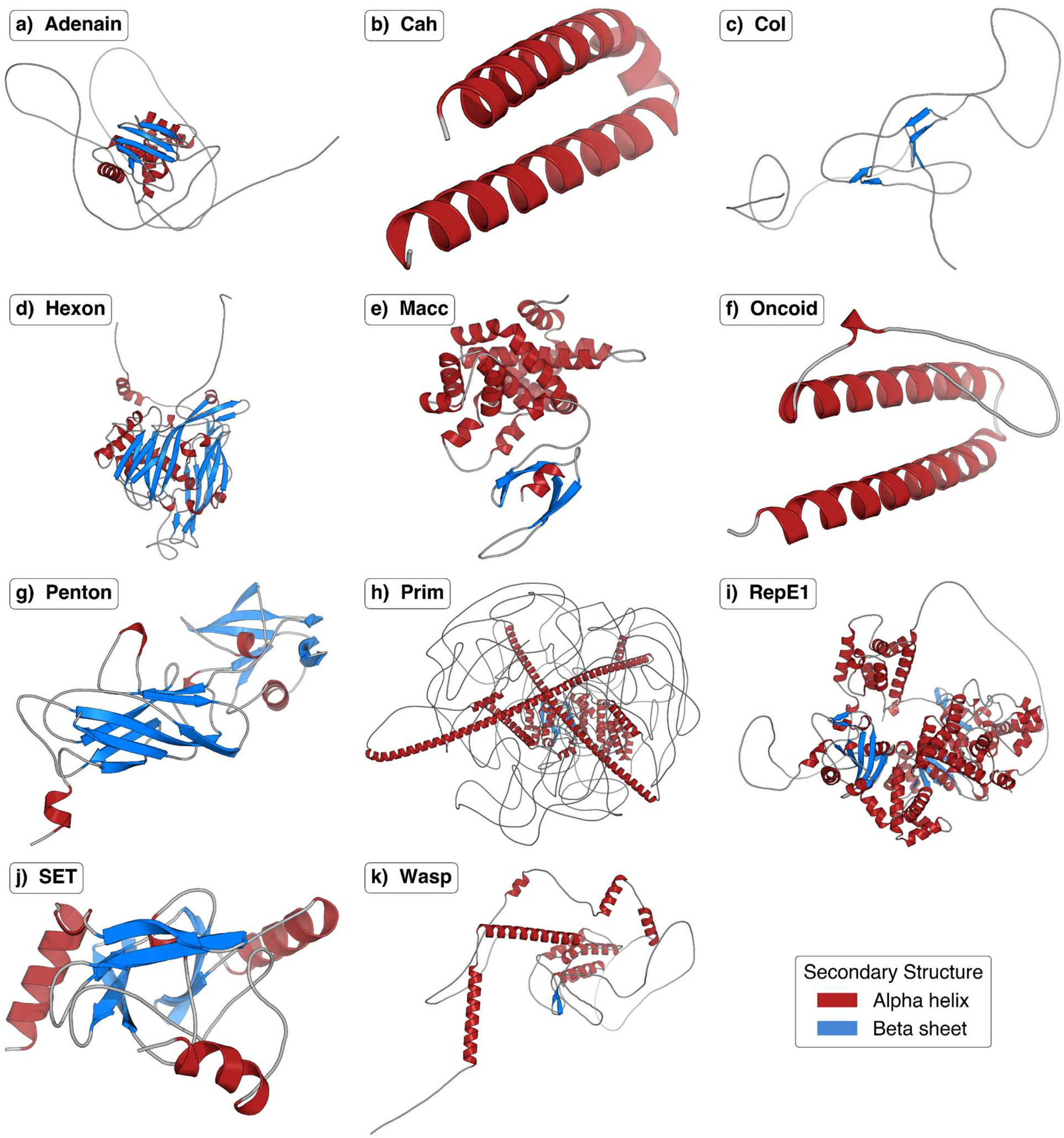

Supplementary Figure 1. AlphaFold-predicted structures for all 7AdoV proteins. The AlphaFold-predicted structures for all 7AdoV proteins are shown. Protein names are displayed on the top left of each panel. Alpha helices are colored red and beta sheets are colored blue. Structures are not shown to scale.

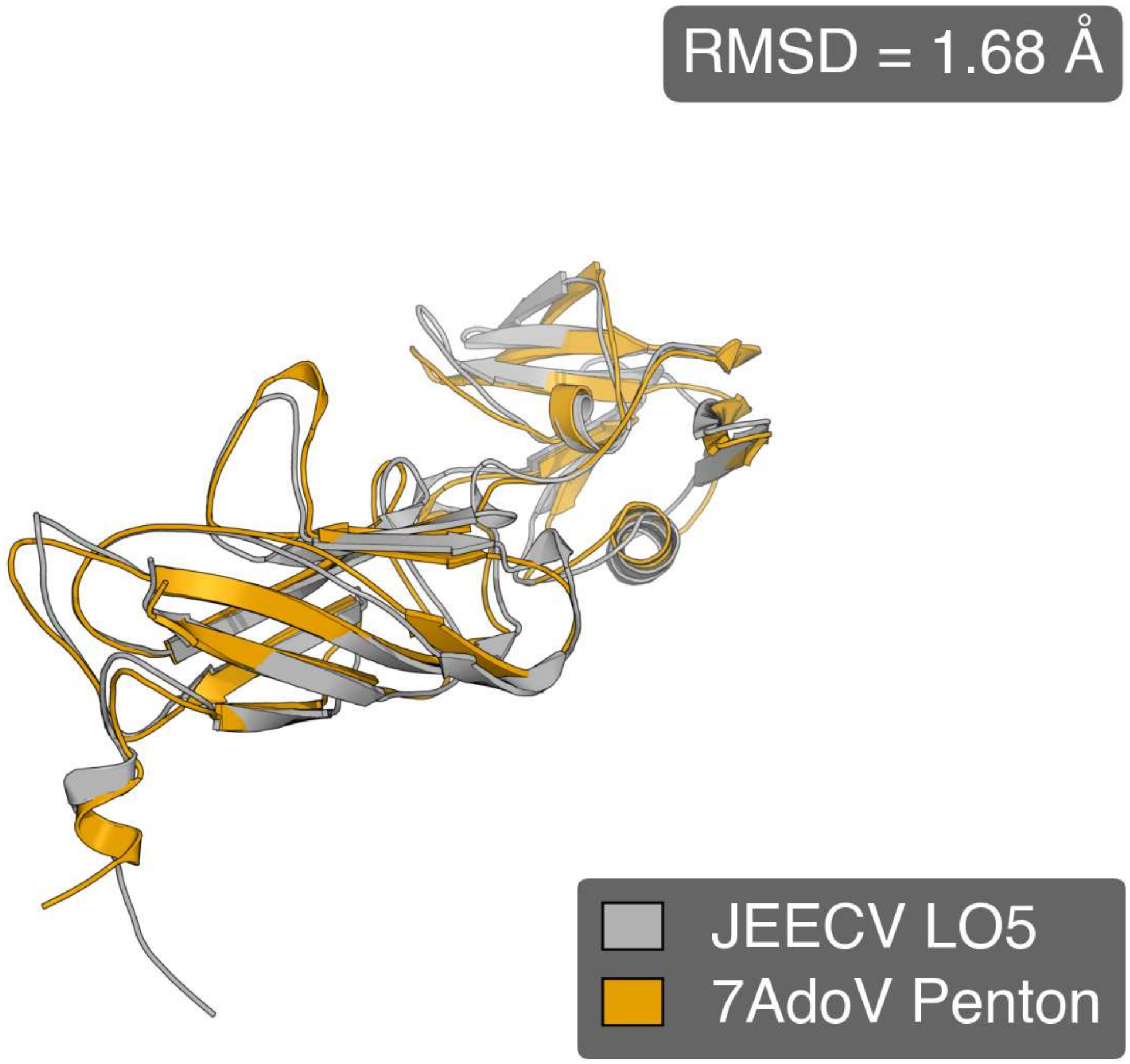

Supplementary Figure 2. Structural comparison of JEECV homolog and 7AdoV Penton. The AlphaFold-predicted structure for the JEECV homolog of Penton was aligned to 7AdoV Penton. Root mean square deviation (RMSD) is shown in Ångstroms for the pairwise comparison. 7AdoV Penton is shown in color.

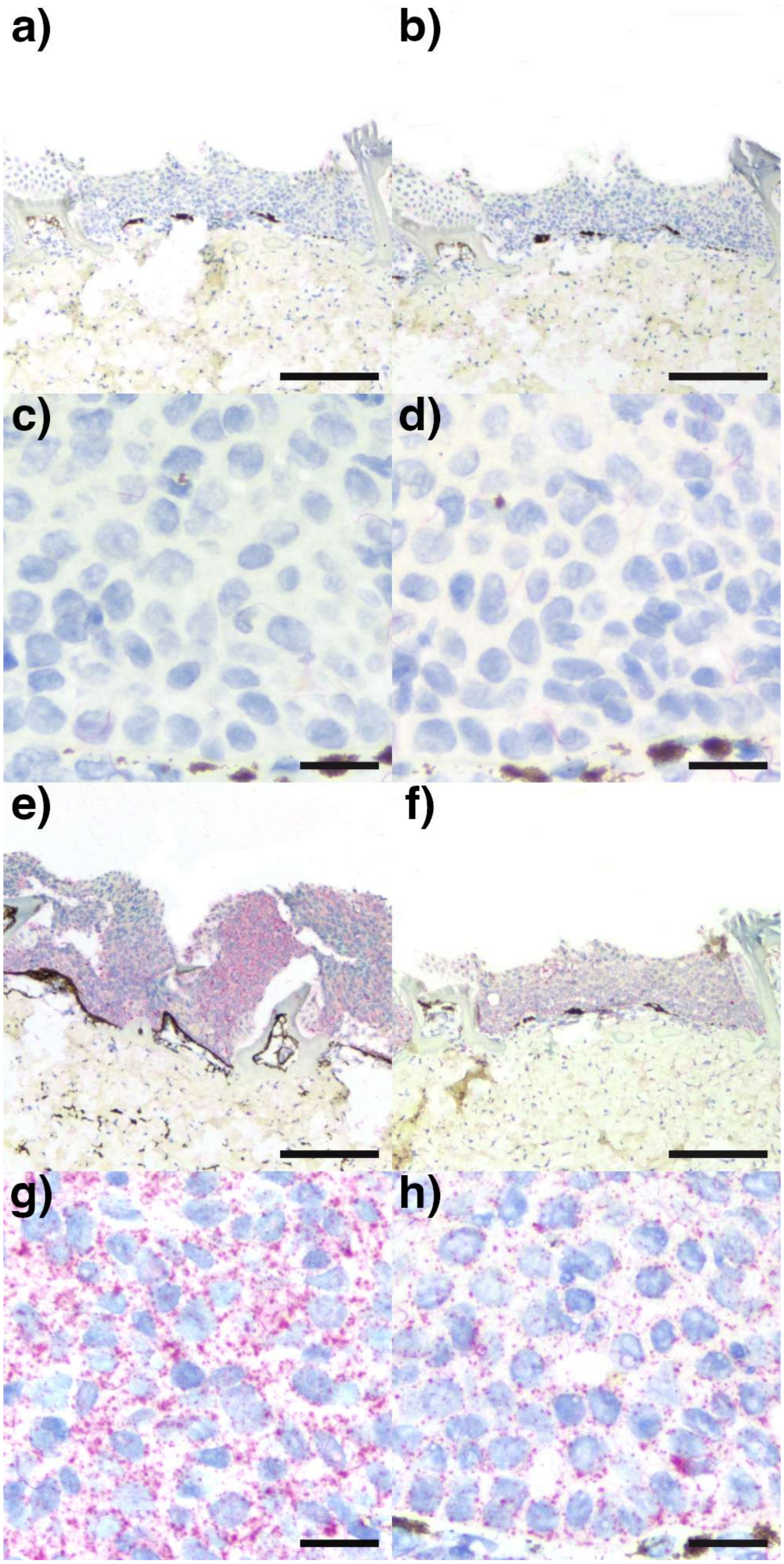
a)
b)
c)
d)
e)
f)
g)
h)

Supplementary Figure 3. Singleplex chromogenic RNAscope *in situ* hybridization performed on non-lesioned and lesioned skin. Low (a, b) and high (c, d) magnification images of histologic sections of non-lesioned skin without epidermal hyperplasia. No hybridization signal, i.e., red labeling, is observed in histologic sections to which the negative control dapB probe was applied (a, c). No hybridization signal is apparent in the epidermis (b) and its constituent squamous epithelial cells (d) after application of the 7AdoV-Wasp probe. Low (e, f) and high (g, h) magnification images of histologic sections of lesioned skin (e, g) with epidermal hyperplasia and non-lesioned skin (f, h) without epidermal hyperplasia to which the sevengill PPIB positive control probe was applied. At low magnification, there is variably intense red hybridization signal throughout the epidermis of both the lesioned (e) and non-lesioned (f) skin, which resolves as punctate red cytoplasmic labeling of epithelial cells at higher magnification in the epidermis of both the lesioned (g) and non-lesioned (h) skin. Hematoxylin counterstain. Scale bar = 200 µm (a, b, e, f) or 20 µm (c, d, g, h).

Supplementary Data 1. HHpred search results for 7AdoV proteins.

Supplementary Data 2. Foldseek search results for AlphaFold-predicted structures.

Supplementary Data 3. Consensus phylogenetic tree in Newick format and GenBank accessions used in tree.
